# Controlling the Synthesis of Polyurea Microcapsules and the Encapsulation of Active Diisocyanate Compounds

**DOI:** 10.3390/polym16020270

**Published:** 2024-01-18

**Authors:** Efterpi Avdeliodi, Anastasia Tsioli, Georgios Bokias, Joannis K. Kallitsis

**Affiliations:** Department of Chemistry, University of Patras, GR-26504 Patras, Greece; chem3577@ac.upatras.gr (E.A.); tsanastasia24@gmail.com (A.T.);

**Keywords:** microcapsules, hexamethylene diisocyanate, isophorone diisocyanate, NCO-compounds, novolac resin

## Abstract

The encapsulation of active components is currently used as common methodology for the insertion of additional functions like self-healing properties on a polymeric matrix. Among the different approaches, polyurea microcapsules are used in different applications. The design of polyurea microcapsules (MCs) containing active diisocyanate compounds, namely isophorone diisocyanate (IPDI) or hexamethylene diisocyanate (HDI), is explored in the present work. The polyurea shell of MCs is formed through the interfacial polymerization of oil-in-water emulsions between the highly active methylene diphenyl diisocyanate (MDI) and diethylenetriamine (DETA), while the cores of MCs contain, apart from IPDI or HDI, a liquid Novolac resin. The hydroxyl functionalities of the resin were either unprotected (Novolac resin), partially protected (Benzyl Novolac resin) or fully protected (Acetyl Novolac resin). It has been found that the formation of MCs is controlled by the MDI/DETA ratio, while the shape and size of MCs depends on the homogenization rate applied for emulsification. The encapsulated active compound, as determined through the titration of isocyanate (NCO) groups, was found to decrease with the hydroxyl functionality content of the Novolac resin used, indicating a reaction between NCO and the hydroxyl groups. Through the thorough investigation of the organic phase, the rapid reaction (within a few minutes) of MDI with the unprotected Novolac resin was revealed, while a gradual decrease in the NCO groups (within two months) has been observed through the evolution of the Attenuated Total Reflectance—Fourier-Transform Infrared (ATR-FTIR) spectroscopy and titration, due to the reaction of these groups with the hydroxyl functionalities of unprotected and partially protected Novolac resin. Over longer times (above two months), the reaction of the remaining NCO groups with humidity was evidenced, especially when the fully protected Acetyl Novolac resin was used. HDI was found to be more susceptible to reactions, as compared with IPDI.

## 1. Introduction

Microencapsulation is a process that allows the controlled loading and release of active compounds [1] and the formation of tiny particles, known as microcapsules (MCs) [2]. The various MCs formed can be inserted as additives to the desired matrix, imparting many added-value functions in various fields of technology, such as in medicine and pharmaceutical as drug delivery systems [3,4], in the textile industry as agents for the release of essential oils or perfumes [5], in coatings as agents that could release substances with anticorrosion effects [6] or in a self-healing capacity that recovers on its own from damage [7,8], etc. In fact, in the case of coatings, microencapsulation currently represents a mainstream research methodology towards effective self-healing properties [9].

In microcapsule technology, controlling the synthesis of the microcapsule shell is a major first goal, while the second goal is the effective encapsulation of active compounds which, when released, will impart new properties to the carrier materials [10]. To this end, various combinations for the formation and control of the microcapsule shell have been extensively studied. Some examples are isocyanate derivatives-amines [11], isocyanate derivatives-alcohols [12] or urea-formaldehyde [13,14], for the formation of the polyurea, polyurethane or urea/formaldehyde shell, respectively.

**Figure 1:**
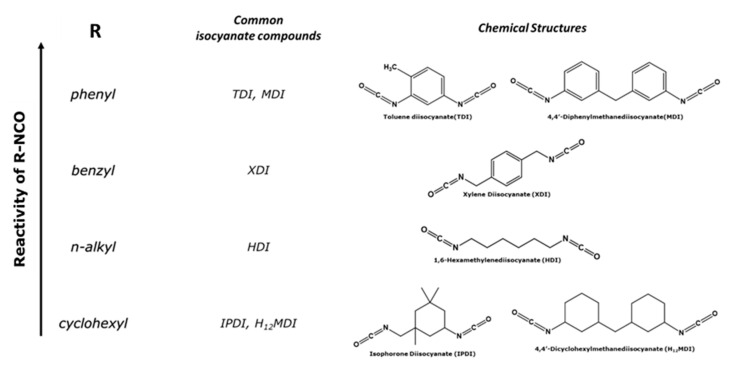
The reactivity order of the most used diisocyanate (R-NCO) compounds.

As it concerns coatings, polyurethanes (PUs) are among the most popular materials for coating applications, since they represent a wide range of uses, as a consequence of their structural versatility [15,16]. Isocyanates (R-NCOs), the main components involved in the synthesis of PUs, are currently becoming, therefore, an important class of industrial chemicals. Isocyanate reactivity is driven by the nature of the main chain, known as the backbone (R), leading to two main groups, namely aliphatic and aromatic isocyanates. When used, aliphatic isocyanates lead to flexible and low tensile strength materials but with increased chain mobility, unlike those synthesized from aromatics isocyanates [17]. Both aliphatic and aromatic R-NCOs are used in different applications, and it is not feasible to interchange them [16] as a consequence of the difference in electrophilicity exhibited by each compound [17]. The reactivity of the R-NCO compound increases with the increasing electrophilicity of the main chain (R), as shown in Figure 1 for diisocyanate compounds. The aromatic R-NCOs are more reactive than the aliphatic counterparts, due to the active hydrogens resulting from the coordination effect, giving free o- and p- substituent positions. Isocyanates can thus react with a variety of functional species, including alcohols, amines, thiols, acids and water. In general, PUs are synthesized through the polycondensation of diisocyanate compounds and various polyols [18]. The high reaction rates of aromatic R-NCOs are often a concern because the selectivity of the reaction is minimized, and it becomes impossible to fully control the polymerization. Indeed, the second monomer reacting with isocyanates plays a key role in the polymerization rate [6,16]. Thus, the reaction of isocyanates with alcohol groups to produce PUs is a thousand times slower than that with amine groups [17]. For this reason, a plethora of synthesis protocols have also been developed combining R-NCOs and amines to prepare polyureas [19].

For the development of MCs as materials for self-healing PUs coatings, the most promising technology is based on microcapsules consisting of polyurea shells [11,20,21]. In this approach, mixtures of diisocyanate compounds are used for a dual purpose, namely (i) for the formation of polyurea shell and (ii) as self-healing agents, taking advantage of the difference in reactivity of the selected R-NCOs. Typically, the highly reactive methylene diphenyl diisocyanate (MDI) (or prepolymers of MDI, the so-called PAPI) is used for polyurea shell formation and the less reactive aliphatic isophorone diisocyanate (IPDI) is encapsulated in the core as a releasable potential self-healing agent [10,11,22].

**Figure 2:**
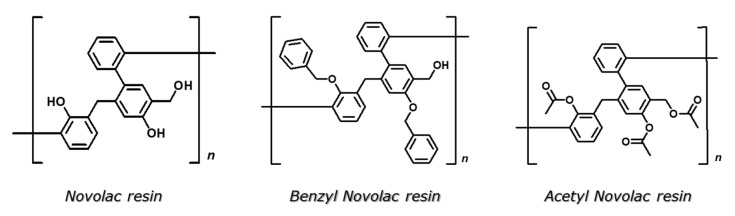
Chemical structure of Novolac resin, Benzyl Novolac resin and Acetyl Novolac resin.

In our research group, we are currently focusing on the development of self-healing materials for waterborne polyurethane (WPUs) coatings [23,24,25]. One of our methodologies is based on the effective encapsulation of active compounds, which, when released, will impart additional functionalities, with the self-healing ability being a first target. Isocyanate compounds, oils and functional polymers are investigated, among others, as encapsulated active compounds. Thus, in our previous work [25], MDI and IPDI were used to prepare core-shell microcapsules through interfacial polymerization in oil-in-water emulsion, using poly(vinyl alcohol) (PVA) as an emulsifier and diethylenetriamine (DETA) as a crosslinking reagent, reacting with MDI. The microcapsules are composed of a PVOH-polyurea composite shell and a liquid core containing IPDI. Aiming at a more effective encapsulation and protection from the hydrolysis of IPDI, a significant novelty in that work was the use of non-isocyanate polymers in the organic phase, namely modified Novolac phenolic resins (Figure 2), as substitutes for the MDI prepolymer (PAPI). Novolac resin has two types of free hydroxyl groups (phenolic and aliphatic hydroxyl groups). The related literature on the modification of Novolac type resins is scarce. The rare publications refer to commercially available phenolic resins with functional hydroxyl groups modified in the form of epoxy rings [26,27] or vinyl esters [27,28]. Thus, in the previous work, both (a) the selective protection of aromatic hydroxyl groups via Williamson benzylation and (b) the total modification of all hydroxyl groups via acetylation were performed, leading to the Benzyl Novolac and Acetyl Novolac resin derivatives, respectively. It was shown that the chemistry of the modified Novolac resin plays a crucial role, with the Acetyl Novolac resin derivative being the most promising, concerning the self-healing ability of WPU coatings containing the respective IPDI-loaded MCs.

The first goal in the present work is to better identify the parameters controlling the formation of microcapsules and, thus, to establish a concrete synthetic protocol for the formation of polyurea shell and the effective encapsulation of active agents. To this end, the influence of parameters like homogenization rate, the composition of organic phases and the MDI/DETA ratio has been evaluated. However, the major goal is to elucidate the importance and possible chemistry involved when the three Novolac resins are present in the core. Thus, we thoroughly investigated just the organic phase and followed the evolution of the NCO content through back-titration and ATR-FTIR spectroscopy. In all stages, we proceeded to a systematic comparative study of two diisocyanate compounds as encapsulated functional agents, namely the cyclic-chain isophorone diisocyanate (IPDI) and the open-chain hexamethylene diisocyanate (HDI). HDI would be a safer alternative for applications since it is characterized by a lower toxicity [24]. On the other hand, HDI is more reactive than IPDI [23], potentially leading to implications as it concerns the stability and possible long-term application of the respective microcapsules.

## 2. Materials and Methods

### 2.1. Materials

Poly(vinyl alcohol) (PVOH, Mw = 130,000 with 88% hydrolysis degree), methylene diphenyl diisocyanate (MDI), hexamethylene diisocyanate (HDI), isophorone diisocyanate (IPDI), diethylenetriamine (DETA), cyclohexanone, acetone, phenol, potassium carbonate, benzyl chloride, sodium chloride, acetyl chloride, dibutyamine and magnesium sulfate-anhydrous were obtained from Merck (Darmstadt, Germany). Formaldehyde was obtained from Petra Chemical Unlimited Corporation (Dallas, TX, USA). Oxalic acid was obtained from Ferak Berlin. Hydrochloric acid, triethylamine, toluene, chloroform and dichloromethane (DCM) were obtained from Alfa Aesar (Ward Hill, MA, USA). Ultrapure water was prepared using an Arium mini water purification system (Sartorius, Göttingen, Germany). The deuterated solvent, DMSO-d6, was obtained from Sigma Aldrich (State of St. Louis, MO, USA). All chemicals in this study were used as received without additional purification. Novolac resin, Benzyl Novolac resin and Acetyl Novolac resin were prepared according to a previously established procedure [25]. The synthesis of Novolac resin was carried out through the polymerization of phenol and formaldehyde monomers in the presence of water, catalyzed by the salt of oxalic acid, at 100 °C for 1.5 h. Benzyl Novolac resin was synthesized through the Williamson reaction using benzyl chloride (80 °C, overnight). Acetyl Novolac resin was synthesized through the acetylation reaction using acetyl chloride (0 °C, overnight). Table 1 summarizes the characteristics of the three resins, as determined through the combination of size exclusion chromatography (SEC) and ^1^H NMR characterization. First, the repeat unit of each oligomer (Figure 2) was identified from the NMR spectra. Then, the equivalents of the hydroxyl groups corresponding to each repeat unit of the oligomers were calculated, while finally, through size exclusion chromatography (SEC), the number-average molar mass (Mn) of the Benzyl Novolac and Acetyl Novolac resins was determined allowing the determination of the number of repeating units and, thus, the calculation of functional hydroxyl groups.

### 2.2. Syntheis of Polyurea Microcapsules (MCs)

The microcapsules were prepared via interfacial polymerization in an oil-in-water emulsion, where the aqueous phase was a PVOH solution in water and the organic phase was a solution in cyclohexanone, containing the desired Novolac resin derivative, MDI, and eventually the desired functional agent (IPDI or HDI). As a typical example, the synthesis of “empty” MCs (not containing IPDI or HDI) was as follows:

A total of 150 mL of 2.5 wt.% PVOH solution was added in a beaker as the phase of the emulsifier using a homogenizer (Kinematica Polytron PT2500E, Malters Switzerland) at an agitation rate of 4000 rpm for 10 min. The organic phase was formed by dissolving 3.7 g (14.8 mmol of isocyanate group) of MDI monomer into a round-bottom flask with 5 mL of cyclohexanone. As the second step, the resin solution was made by dissolving 5 g of Acetyl Novolac resin into a beaker with 10 mL of cyclohexanone under magnetic stirring at room temperature (RT). Then, the resin solution was added into the round-bottom flask of isocyanate mixture under argon and stirred at RT for 3 min. At that moment, the organic phase was poured into the solution of the emulsifier under homogenization. After 5 min, for the creation of the microcapsules’ shell, 1.02 g (29 mmol of amino groups) of DETA dissolved in 30 mL of deionized water was slowly poured into the emulsion to start the interfacial polymerization. Subsequently, the emulsion polymerization reaction was completed after 3 h using a homogenizer at a range of agitation rates (rpm) at RT. The microcapsules were filtered by using filter paper and left overnight at RT. The resulting capsules were washed by redispersing them in deionized water. The dispersion was left to stand overnight; the precipitated capsules were collected and air-dried at RT for 2 days before further use.

The same basic protocol was applied for all MCs described in the present work, modified accordingly, for MCs containing Novolac resin or Benzyl Novolac resin, as well as IPDI or HDI. The final products are coded as A-B MCs, where A is I or H for MCs containing IPDI or HDI, respectively, while B is Nov, Bnz or Ac for MCs containing Novolac resin, Benzyl Novolac resin or Acetyl Novolac resin, respectively. The synthesis details for the materials prepared are summarized in Table 2.

### 2.3. Characterization of MCs

To characterize the MCs, 0.2 g of the final product were crushed in an agate mortar and sonicated in acetone for 60 min. The suspension was centrifuged at a speed of 6500 rpm for 15 min. This process was repeated twice and, finally, the supernatant and the precipitate were collected. The obtained precipitate, considered the shell, was dried at room temperature (RT), while the core was stored as the acetone solution from the microcapsules’ extraction. Both the initial MCs and the shell were characterized through ATR-FTIR, while the NCO wt.% content of the core was quantified through back-titration applying the ASTM D2572 standard [29].

### 2.4. Study of the Organic Phase

Organic solutions with a similar composition (i.e., containing cyclohexanone, MDI, Novolac derivative, as well as HDI or IPDI) as that used for the preparation of MCs were prepared for this study. Thus, for the mixtures containing HDI, 1 g (8 mmol of isocyanate group) and 3.36 g (40 mmol of isocyanate group) of HDI were dissolved in 5 mL of cyclohexanone. At the same time, 5 g of the desired Novolac derivative were dissolved in 10 mL of cyclohexanone under magnetic stirring at RT. Then, the resin solution was added to the isocyanate mixture and stirred at RT for 3 min. For the mixtures containing IPDI, 5 g (45 mmol of isocyanate group) of IPDI was used, instead of HDI.

In line with the abbreviations used for MCs in Table 2, the mixtures will be denoted H-NOV, H-BNZ and H-AC in the case of HDI, and I-NOV, I-BNZ and I-AC in the case of IPDI. The evolution of the mixtures was followed visually (or through optical density measurements), as well as through ATR-FTIR spectroscopy. The NCO wt.% content was quantified through back-titration applying the ASTM D2572 standard.

### 2.5. Characterization

FTIR spectra were collected using an Alpha II spectrometer (Bruker) (Billerica, MA, USA), with a diamond ATR crystal. All spectra were recorded in the range of 4000–400 cm^−1^, with an average of 34 scans and a spectral resolution of 4 cm^−1^.

The morphology and size distribution of the obtained MCs were determined using scanning electron microscopy (SEM) using a Zeiss ZUPRA 35 VP-FEG instrument (Jena, Germany), operating at 5–20 keV. An aqueous dispersion of MCs was spread on a Si wafer, allowed to dry and coated with a conductive Au film through sputtering.

According to the ASTM D2572 standard [20], isocyanate functional groups of samples are transformed into urea using dibutylamine solution in toluene followed by the titration of the excess amine with standardized hydrochloric acid solution [30]. All organic solvents were dry to avoid the reaction between NCO groups and residual water. The relative percentage of the NCO compound can be calculated from the equations, which are presented in Section S1. Titrations were performed in triplicate, providing a mean error of ±2%.

^1^H NMR spectroscopy was applied to elucidate the chemical composition of the synthesized resins. Novolac, Benzyl-modified Novolac and Acetyl-modified Novolac resins were dissolved in DMSO-d6 for 1H NMR characterization. Then, 1H NMR spectra were recorded on a Bruker AVANCE DPX 400 MHz spectrometer (Billerica, MA, USA).

SEC measurements were performed at 25 °C and a 1 mL/min flow rate with CHCl3 as eluent for modified resins. A Polymer Lab chromatograph equipped with Ultra Styragel columns (104, 500 Å), a UV detector (254 nm) and a refractive index detector were used. Standard polystyrene (PS) samples were used to calibrate the columns.

The optical density at 500 nm was measured using a Hitachi U-1800 UV−Vis spectrophotometer (Dallas, TX, USA) equipped with a circulating water bath, set at 25 °C.

## 3. Results

### 3.1. Synthesis and Characterization of Microcapsules

**Figure 3:**
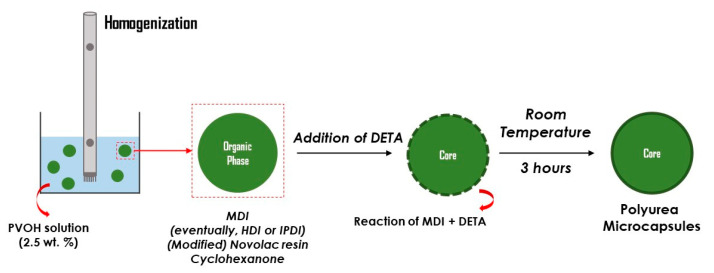
Schematic drawing of the synthesis of polyurea microcapsules via interfacial oil-in-water emulsion polymerization.

The polyurea microcapsules were synthesized via interfacial polymerization in an oil-in-water emulsion, as schematically shown in Figure 3. The aqueous phase was a 2.5 wt.% PVOH solution in water [31,32]. For the organic phase, cyclohexanone was used as a solvent. This phase also contains MDI (for the formation of the microcapsule shell) and eventually a (modified) Novolac resin, as well as the potential encapsulated active agent, namely IPDI or HDI. During emulsification, an interconnected network of hydroxyl groups is formed at the interface between the aqueous and organic phases, which can aid the microcapsules to form hydrogen bonds [11,33]. When diethylenetriamine (DETA) is introduced, the primary polymerization reaction between the amine functional groups of triamine in the aqueous phase and the isocyanate functional groups of MDI in the organic phase takes place at the oil/water interface to produce a polymeric polyurea shell surrounding the organic droplets.

As a first step, the synthetic protocol for the preparation of empty microcapsules was investigated. With the term “empty”, a protocol not involving the encapsulation of any active compound is meant. Thus, the organic phase consists of the solvent, the MDI and, eventually, the Acetyl Novolac resin. A homogenization rate of 4000 rpm was selected for these studies.

**Figure 4:**
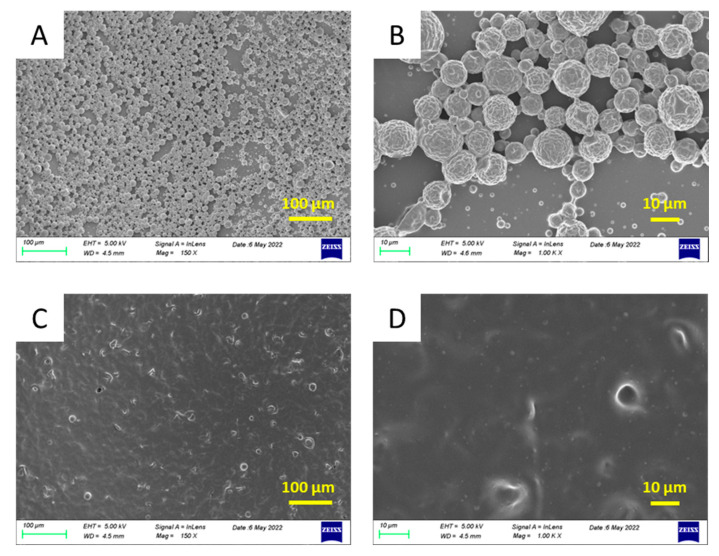
Morphology of “empty” microcapsules prepared using Acetyl Novolac resin. The homogenization rate was 4000 rpm. Ratio of MDI/DETA monomers was (**A**,**B**) 1.5/1 and (**C**,**D**) 1/1.

**Figure 5:**
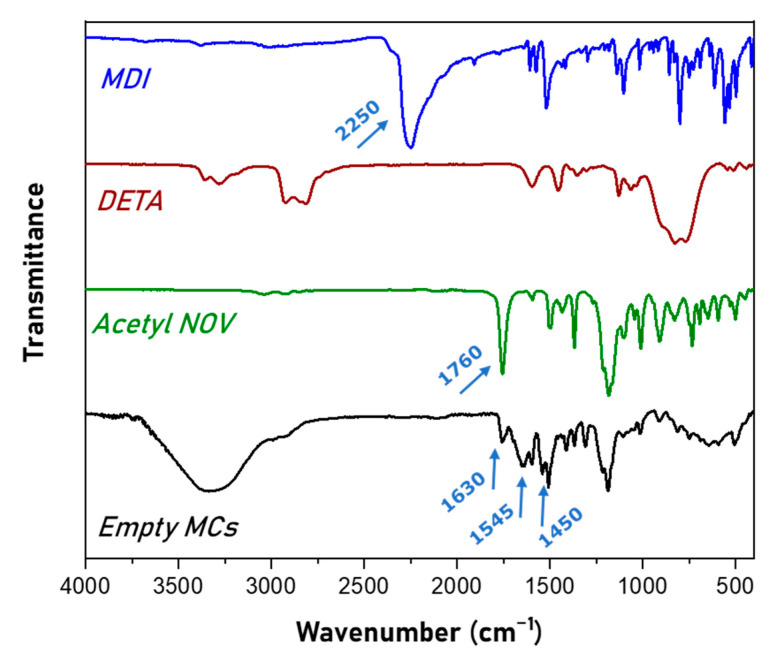
FTIR-ATR spectra of MDI, DETA, Acetyl Novolac resin and empty microcapsules formed at MDI/DETA ratio equal to 1.5/1.

Since Acetyl Novolac resin is fully protected and it does not contain any -OH groups, it is inert towards MDI. This organic phase is, thus, appropriate to test the influence, if any, of the ratio of the reagents forming the shell of the microcapsules, the MDI and the DETA. Two MDI/DETA ratios were tested: 1/1 and 1.5/1. In the first case, the NCO groups of the MDI were equivalent, with the total of the primary and secondary amine groups of the DETA, while just the primary amine groups of the DETA were equivalent with the NCO groups of the MDI in the second case. As seen in the SEM images in Figure 4A,B, microcapsules with a size ~10 μm were obtained when the MDI/DETA was 1.5/1. In contrast, when the MDI/DETA ratio was 1/1, the microcapsules (if any) were hardly observed over a uniform polymeric substrate. These observations indicate that all amine groups of DETA should participate in the formation of the capsule, thus creating a tightly crosslinked polyurea shell. This is in agreement with the fact that the characteristic peaks of DETA do not appear in the spectrum of “empty” MCs, as will be shown in Figure 5. When the MDI/DETA ratio is 1.5/1, one third of the amine groups are in excess and do not react. In combination with the fact that DETA possesses two primary amine groups and a (more hindered) secondary amine group, this probably leads to mostly linear or loosely cross-linked polymer chains. As a consequence, this allows the polymer to precipitate, instead of forming a well-structured microcapsule.

The empty microcapsules with MDI/DETA ratios at 1.5/1 were further characterized through FTIR-ATR. The spectrum is compared in Figure 5 with the spectra of MDI, DETA and Acetyl Novolac resin. In the spectrum of microcapsules, the characteristic peaks at 1630 cm^−1^, 1545 cm^−1^ and 1450 cm^−1^ of the carbonyl group (C=O), the amino group (N-H) and the C-N bond are observed, indicating the formation of the polyurea shell. This is further supported by the fact that the peak at 2250 cm^−1^, attributed to the –NCO stretch, is not observed in the spectrum of MCs. Moreover, the presence of Acetyl Novolac Resin in the microcapsules is verified by the observation of the characteristic peaks at 1760 cm^−1^, attributed to carbonyl group, and at 1306 cm^−1^, attributed to the C-O bond of the ester formed after the acetylation of Novolac resin.

**Figure 6:**
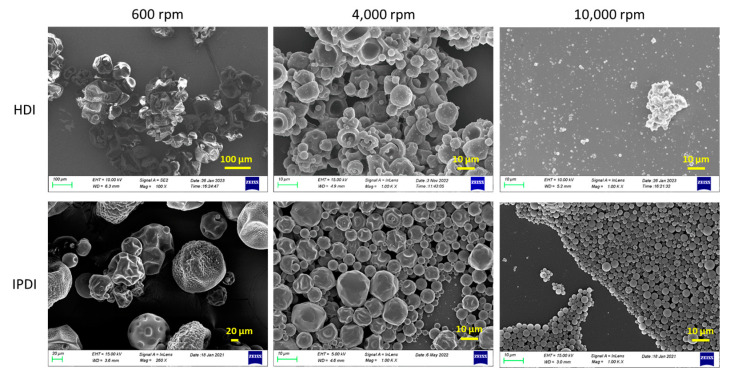
SEM images of H-Bnz MCs and I-Bnz MCs obtained at a homogenization rate of 600 rpm (**left**), 4,000 rpm (**center**) or 10,000 rpm (**right**).

Another important parameter of the synthesis protocol tested was the influence of the homogenization rate. Three homogenization rates, namely 600 rpm, 4000 rpm and 10,000 rpm, were tested for HDI- and IPDI-loaded MCs. The morphology and size distribution of the capsules were evaluated using scanning electron microscopy (SEM). For example, the SEM images of the I-Bnz MCs and H-Bnz MCs are shown in Figure 6. It is clear that the homogenization rate offers a tool to effectively control the size of the MCs formed. As expected [34,35,36], the sizes of the MCs decrease with increasing the homogenization rate. Thus, objects of the order of 100 μm are obtained at the lower homogenization rate of only 600 rpm. The size decreases drastically (10–20 μm) for the intermediate homogenization rate (4000 rpm) and becomes about 1 μm when the homogenization rate is 10,000 rpm. Moreover, in most cases, more or less well-formed spherical objects are observed, especially for the I-Bnz MCs and higher homogenization rates. Similar trends were also observed for IPDI- or HDI-loaded MCs formed using Novolac or Acetyl Novolac resin. It should be mentioned that the shell deformation of the MCs, observed in some SEM images, could result from the sample preparation procedure, including drying and metal sputtering.

**Figure 7:**
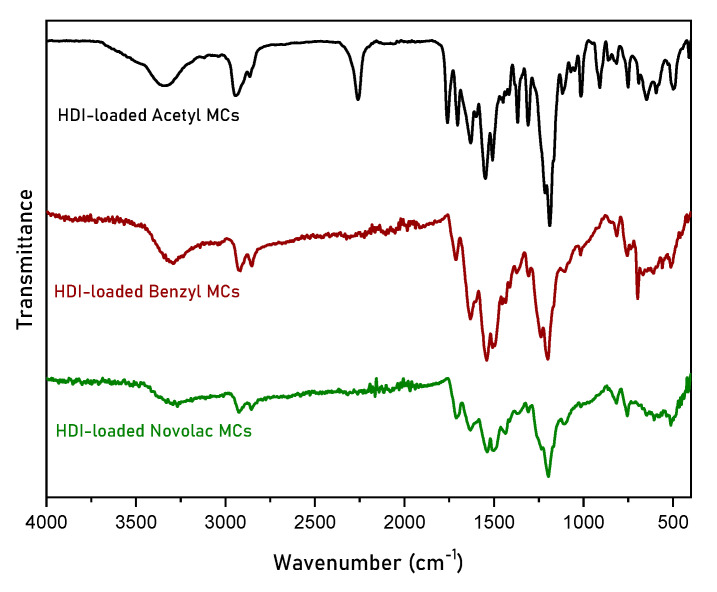
ATR-FTIR spectra of HDI-loaded MCs obtained at a homogenization rate of 4000 rpm, using Acetyl Novolac resin, Benzyl Novolac resin and Novolac resin.

All the MCs prepared at 4000 rpm were further characterized through ATR-FTIR spectroscopy. The characterization of the HDI-loaded MCs is shown Figure 7. The characteristic signals of the polyurea shell at 1630 cm^−1^, 1545 cm^−1^ and 1450 cm^−1^ of the carbonyl group (C=O), the amino group (N-H) and the C-N bond are observed in all spectra. In addition, the H-Bnz MCs made with Benzyl Novolac resin show the characteristic peak of the monosubstituted benzene ring at 700 cm^−1^, while the H-Ac MCs made with Acetyl Novolac resin show the characteristic peak of the resin at 1760 cm^−1^ for the carbonyl group of the ester synthesized after acetylation. Finally, the characteristic peak at 2250 cm^−1^ (–NCO stretch) is clearly observable in the case of the H-Ac MCs, indicating that these MCs are rich in HDI. In contrast, this peak is not observed in the cases of the H-Nov and H-Bnz MCs, suggesting that the HDI content of these MCs is now considerably lower.

**Figure 8:**
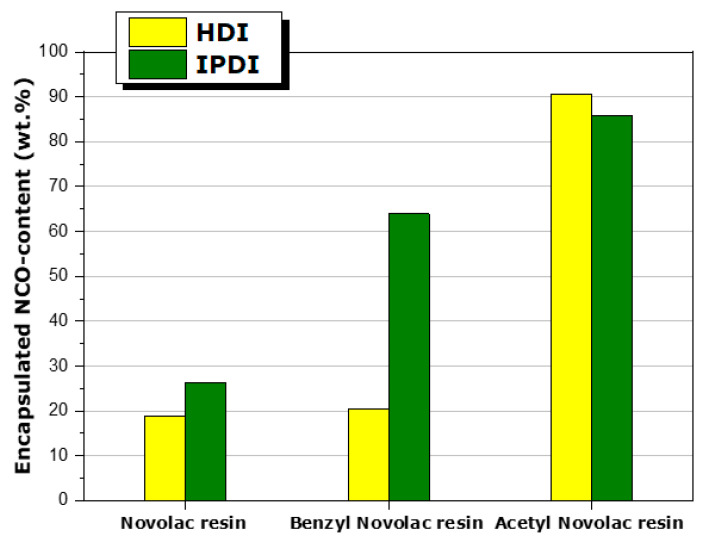
The encapsulated NCO-content for the three types of capsules containing HDI or IPDI.

A similar trend, as it concerns ATR-FTIR spectra, is also observed for the IPDI-loaded MCs [37]. However, it was verified that the MCs indeed do contain IPDI after the extraction of the core using acetone. In fact, the extraction of the core, in combination with the back-titration of the NCO groups, applying the ASTM D2572 standard, permits the quantification of the encapsulated isocyanate content of the MCs. According to this standard, the isocyanate functional groups are modified to urea using an excess of dibutylamine diluted in dry toluene. After the reaction, the excess amine is titrated with a standardized aqueous hydrochloric acid solution [30]. The three types of HDI- or IPDI-loaded MCs, prepared at a homogenization rate of 4000 rpm, were titrated about 40 days after preparation and recovery in solid form. The results are presented in Figure 8. In the case of the Ac MCs, where the fully protected Acetyl resin was used, almost all NCO content was detected, suggesting that both HDI and IPDI remain practically intact and entrapped successfully, without being hydrolyzed by the presence of water during emulsion polymerization. In fact, the values are about 85–90%, i.e., somewhat lower than 100%, apparently due to the consumption of the NCO groups (originating from MDI) for the formation of the shell of the MCs. In contrast, for the Nov MCs, the NCO content is quite low (20–25%). While a reason for this could be the possible release and reaction of the active agents with functional species present (humidity, for instance) during storing, the most probable is that isocyanate groups are consumed through the reaction with the abundant hydroxyl groups of Novolac resin. Finally, an intermediate behavior is observed in the case of the Bnz MCs. Thus, an intermediate NCO content of about 65% is detected for the I-Bnz MCs, whereas the NCO content is very low, about 20%, for the H-Bnz MCs, comparable to that found in the H-Nov MCs.

### 3.2. Study of HDI- or IPDI-Loaded Organic Phase

The previous findings indicate that the active isocyanate ingredients are possibly involved in reactions with the unprotected hydroxyl groups of (modified) Novolac resins. Moreover, the relative reactivity and stability of the two isocyanate compounds, HDI and IPDI, may play an important role, since open-chain aliphatic isocyanate derivatives (HDI) are stated to be more active than those of the cyclic chain isocyanate derivatives (IPDI) [17,30]. For this reason, we proceeded to study the mixtures of just the organic phases for both isocyanate derivatives, using all three types of Novolac resins, over time. Six mixtures were prepared in proportions and ingredients according to Section 2.4 (Table 2). The mixtures were qualitatively studied visually and through optical density measurements; they were characterized through ATR-FTIR spectroscopy, while the NCO wt.% was quantified through titration, applying the ASTM D2572 standard. Table 3 shows the amount of equivalents contained in each mixture for the characteristic groups of the hydroxyl groups of the Novolac resin and the NCOs of each isocyanate derivative.

Representative photos of the appearance of the mixtures, just after preparation and two weeks later, are shown in Figure S1. It is observed that the organic phases containing the fully protected Acetyl resin remain practically transparent for at least a 2-week period. In the case of the partially protected Benzyl resin, a slight turbidity is observed (for the mixture I-BNZ) after two weeks. In contrast, the mixtures with the unprotected Novolac resin, I-NOV and H-NOV, shortly turn strongly turbid. These observations offer the first indications that the hydroxyl groups of Novolac resin (and, possibly, Benzyl Novolac resin) are involved in reactions with the NCO groups of the diisocyanate compounds present in these mixtures.

**Figure 9:**
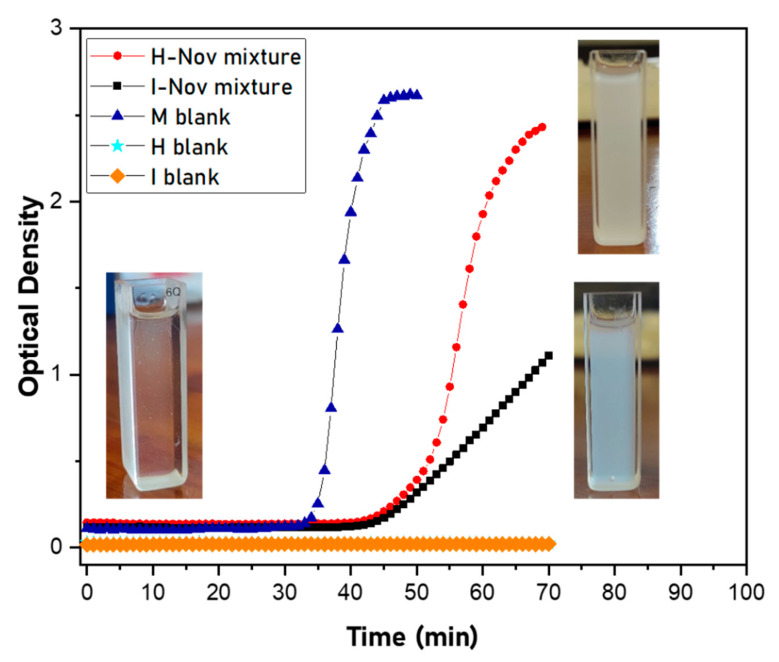
Evolution of the optical density of H-NOV and I-NOV mixtures with time. Photos of the mixtures are inset in the before (clear) and after (cloudy) diagram over time. The respective evolution of blank mixtures: M-blank (without the presence of IPDI or HDI), H-blank (mixture with HDI, without MDI) and I-blank (mixture with IPDI, without MDI). H-blank data (siel color) are “hidden” behind of the I-blank data (orange color).

The evolution of the turbidity of I-NOV and H-NOV mixtures was studied in more detail through monitoring the optical density of the mixtures at 500 nm (Figure 9). It should be noted that, similarly to the composition of the organic phases for the MCs’ preparation, these mixtures also contain a small amount (about 4 wt.%) of MDI, apart from HDI or IPDI. To obtain a better understanding, therefore, the simple organic phases, containing just MDI, IPDI or HDI at identical concentrations as those in the I-NOV and H-NOV mixtures, were also investigated. These simple mixtures are denoted as MDI-NOV, IPDI-NOV and HDI-NOV, respectively. In the case of the organic phase containing only MDI or MDI-NOV, a strong turbidity is observed about half an hour after mixing, indicating the formation of an insoluble product, probably due to the reaction with Novolac resin. In contrast, the organic phases containing only IPDI or HDI, IPDI-NOV or HDI-NOV remain transparent for a longer time. In fact, we have verified that after 5 days the HDI-NOV mixture formed an insoluble product, whereas the IPDI-NOV mixture was still transparent. These observations suggest that the reaction of HDI or IPDI with Novolac resin are slow (as compared with the behavior of MDI) or lead to soluble products. This is further supported by the evolution of ATR-FTIR spectra and the titration of the NCO groups, as will be shown in the following studies. Finally, the mixtures containing MDI and IPDI, or HDI, I-NOV or H-NOV, turn turbid somewhat later than the MDI-NOV mixture, while the turbidity increase in the I-NOV mixture is gradual compared with the behavior of the H-NOV mixture. Though this behavior is not fully understood, the competition of the reaction of Novolac with IPDI or HDI and MDI (in the I-NOV or H-NOV organic phases, respectively) is probably the reason for the observed behavior.

**Figure 10:**
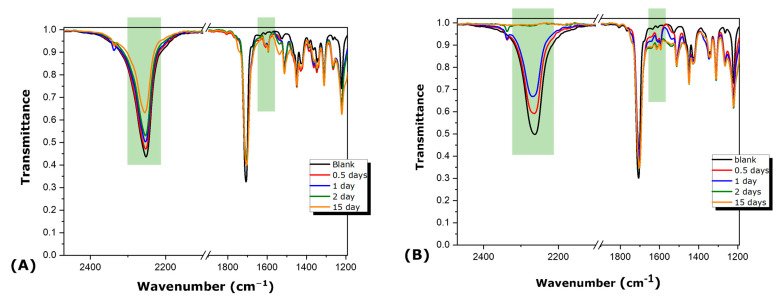
Evolution of ATR-FTIR spectra of (**A**) I-NOV (**B**) H-NOV mixture with time. Green highlights at 2250 and 1596 cm^−1^ represent the NCO peak and NH peak, respectively.

All mixtures were also characterized through ATR-FTIR as a function of mixing time. In Figure 10 characteristic spectra for I-NOV and H-NOV mixtures are shown. It is clearly observed that the characteristic peak at 2250 cm^−1^, attributed to NCO group, gradually decreases with time. In addition, a small peak at 1596 cm^−1^ appears and gradually strengthens with time. This peak can be attributed to NH group, probably arising from urethane units formed through the reaction of NCO with the hydroxyl groups of Novolac resin.

**Figure 11:**
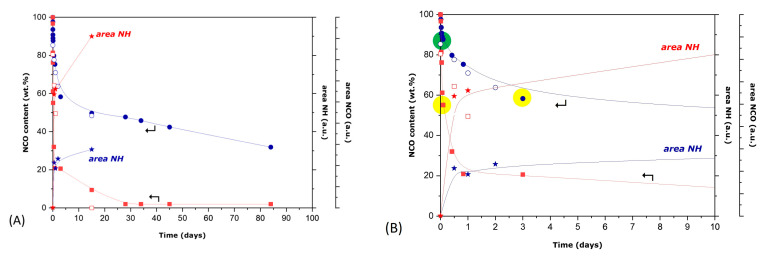
Decrease in NCO (wt.%) in the I-NOV and H-NOV mixtures in the presence of the active hydroxyl groups of Novolac resin for (**A**) a three month period and (**B**) the first ten days. The red color corresponds to the H-NOV mixture, while the blue color corresponds to the I-NOV mixture. The NCO content (from the titrations) is presented with a solid square (I-NOV) or circle (H-NOV). The NCO areas and NH areas are shown with open square (I-NOV) or open circle (H-NOV) and star symbol, respectively. The NCO consumption of MDI is shown in green marking and the Novolac aliphatic OH consumption is shown in yellow marking.

While the peak at 1596 cm^−1^ is weak and can be just qualitatively evaluated, the variation in the area of the peak at 2250 cm^−1^ is significant and can be used to quantify the NCO consumption over time, considering that the area of this peak in the very first spectrum is representative of the number of NCO groups that are initially present in the mixture. In addition, the consumption of NCO groups was also determined through titration. The results are presented in Figure 11, where they are compared with the respective results obtained from the evolution of the ATR-FTIR spectra. As seen, the results from both techniques are in rather good agreement, showing the same trends for the two mixtures. The green circles in these results indicate the points for full MDI consumption (assuming that they are first consumed). Moreover, the yellow circles indicate the points of full consumption of the aliphatic hydroxyl groups, originating from Novolac resin. In the case of the I-NOV mixture, a rapid consumption is initially observed (for about 5 days), while NCO groups are consumed at a much lower rate for higher mixing times (up to several months). As seen, the point of rate change coincides well with the consumption of the aliphatic hydroxyl groups of Novolac resin. This is an indication that the aliphatic hydroxyl groups are initially consumed, while the phenolic groups of Novolac resin are consumed at higher mixing times. The slow rate of consumption of the phenolic groups could be due to steric hindrance reasons. In line with the higher reactivity of HDI, as compared with IPDI, the reactions are much more rapid in the case of the H-NOV mixture. Thus, the consumption of the aliphatic hydroxyl groups of Novolac resin takes place within the first two hours, while the content of the NCO groups continues to rapidly decrease for about 1 day (apparently suggesting the consumption of the phenolic groups, also). The NCO content continues to gradually decrease up to day 25, when it becomes practically zero, indicating the further consumption of the phenolic groups of Novolac resin. Nevertheless, at such high mixing times, the possibility of the reaction of both diisocyanate compounds (IPDI or HDI) with moisture should not be neglected. The evolution of the area of the NH peak, indicating the formation of polyurethane groups, follows an inverse trend, as compared with the evolution of NCO consumption. Moreover, in agreement with the variation in reactivity of HDI and IPDI, a much higher NH signal is detected in the case of the H-NOV mixture as compared with the signal detected for the I-NOV mixture. Finally, it should be noted that the consumption of the NCO groups from MDI is just a small part of the whole change taking place probably at the very first mixing period (as also suggested from the turbidity study, Figure 9). This rapid reaction and precipitation may interfere with the interfacial polymerization reaction of MDI with DETA. As a matter of fact, this possibly explains the observation that the microparticles formed in the presence of Novolac resin are strongly deformed and far from spherical, especially in the case of HDI (Figure S2).

**Figure 12:**
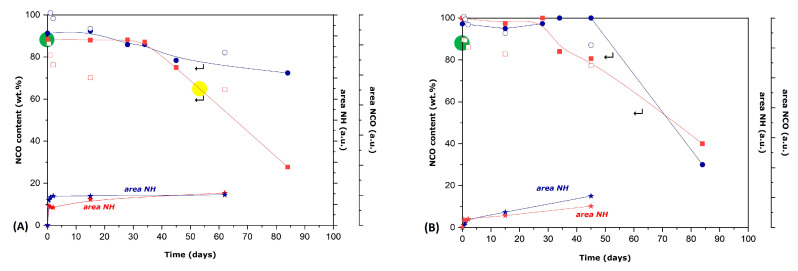
(**A**) Decrease in NCO (wt.%) in the I-BNZ and H-BNZ mixtures in the presence of the active hydroxyl groups of Benzyl Novolac resin with time. (**B**) Decrease in NCO (wt.%) in the I-AC and H-AC mixtures in the presence of Acetyl Novolac resin with time. The red color corresponds to the H-BNZ or H-AC mixtures, while the blue color corresponds to the I-BNZ or I-AC mixtures. The NCO content (from the titrations) is presented with a solid square (I-BNZ and I-AC) or circle (H-BNZ and H-AC). The NCO areas and NH areas are shown with open square (I-BNZ and I-AC) or open circle (H-BNZ and H-AC) and star symbol, respectively. The MDI’s NCO consumption is shown in green marking and the Benzyl Novolac aliphatic OH consumption is shown in yellow marking.

Similar ATR-FTIR and titrations studies were also performed for the mixtures of IPDI and HDI with Benzyl Novolac and Acetyl Novolac resin. The evolution of the ATR-FTIR spectra is presented in Figures S3 and S4, while the consumption of the NCO groups, as determined by both methods, is shown in Figure 12A,B, for the mixtures with Benzyl and Acetyl Nolovac resin, respectively. It is important to remember that Benzyl Novolac resin possesses only aliphatic hydroxyl groups. Thus, in Figure 12A, a gradual decrease in the NCO groups is seen for the I-BNZ mixture. Up to day 85, about 30 wt.% of the initial NCO amount was consumed, corresponding to a quantity somewhat lower than the aliphatic OH groups (11.7 meq OH) of the Benzyl Novolac resin. For the H-BNZ mixture, an analogous decrease in NCO groups was noted up to day 45, corresponding to the full consumption of all the OH groups of Benzyl Novolac resin (12.1 meq OH). However, in this case, the consumption of the NCO groups continued for longer times. Since all the OH groups were consumed, this further NCO decrease probably resulted from the reaction with humidity. In fact, the influence of humidity is more evident for the studies with the Acetyl Novolac resin (Figure 12B). Indeed, a significant decrease in NCO content is observed after day 50 for the I-AC mixture and after day 30 for the H-AC mixture. Since Acetyl Novolac resin is fully protected and does not possess any hydroxyl groups, humidity is now the only possible source for the hydroxyl groups reacting with the NCO groups. To further verify these observations, additional experiments are planned to follow the mixtures under controlled humidity conditions.

It should be mentioned that the overall behavior observed in this section is in qualitative agreement with the results shown in Figure 8, reporting the NCO content of the respective MCs after a period of 40 days. Indeed, the NCO content of MCs is consistent with the reaction of the encapsulated agent with the hydroxyl groups of the three Novolac resins. In addition, the decreased value observed for the H-Bnz MCs, as compared with the I-Bnz MCs, is in qualitative agreement with the faster NCO consumption in the case of the H-BNZ mixture as compared with the I-BNZ mixture, as a consequence of the higher reactivity of HDI. In fact, though not fully understood, the hydrolysis of HDI due to humidity has probably already occurred in the case of H-Bnz MCs, as compared with the I-Bnz MCs. The possible hydrolysis of both HDI and IPDI in the presence of humidity is more clearly evidenced over longer times when the fully protected Acetyl Novolac resin is used (Figure 12B). This has important consequences to the stability and long-term application of MCs. Indeed, as shown elsewhere [38], the NCO content of I-Ac MCs decreases rapidly when the microcapsules are dispersed in aqueous solution, as compared with the respective behavior when they are stored at room conditions. In fact, the decrease in NCO content due to reaction with humidity has been reported for other similar MCs after long-time storing [39,40].

## 4. Conclusions

The conditions for the successful encapsulation of two potential self-healing agents, namely IPDI and HDI, in polyurea microcapsules (MCs) have been identified in the present work. The polyurea shell of the microcapsules is formed through the interfacial oil-in water emulsion polymerization of MDI and DETA. The core of the microcapsules, apart from the solvent, MDI and the self-healing agent, also contains a Novolac resin, either unprotected or protected (in the form of partially protected Benzyl Novolac resin or fully protected Acetyl Novolac resin). As a general conclusion, the application of a homogenization rate of 4000 rpm and an MDI/DETA ratio leading to the reaction of all available amine groups are crucial parameters for the successful formation of MCs with a size of 10–20 μm and the effective encapsulation of active agents. In addition, from the NCO content of the MCs, the reaction with available hydroxyl groups of the Novolac resin used is evidenced. To obtain a deeper insight of the reactions involved, the evolution of the respective organic phases was explored through turbidimetry, ATR-FTIR spectroscopy and NCO titration. Our results support the faster and more quantitative reaction of HDI with available OH groups, as compared with IPDI. In addition, the possible reaction with humidity has been evidenced.

From the present study, valuable information can be derived for the development of polyurea MCs using Novolac resins as an adequate substrate to control the properties of the core. This information is important for the rational design of effective encapsulation in MCs, IPDI or HDI when self-healing applications are targeted. However, this information could also be useful for the effective encapsulation of alternative active agents when other applications are envisaged.

## Figures and Tables

**Table 1:** Characterization results for the three Novolac resin derivatives.

Product	Number of Repeating Units	Mn	Total mol of OH	mol of Phenolic OH	mol of Aliphatic OH
Novolac resin	4	921	12	8	4
Benzyl Novolac resin	4	1700	4	-	4
Acetyl Novolac resin	4	1300	0	-	-

**Table 2:** Feed composition of synthesized microcapsules.

Type of MCs	MDI (g)	IPDI (g)	HDI (g)	Type of Resin	Resin (g)	Cyclohexanone (mL)	DETA (g)
Empty MCs	3.7	-	-	Acetyl Novolac	5	15	1.02
I-Nov MCs	1	5	-	Novolac			
I-Bnz MCs				Benzyl Novolac			
I-Ac MCs				Acetyl Novolac			
H-Nov MCs	1	-	3.36	Novolac			
H-Bnz MCs				Benzyl Novolac			
H-Ac MCs				Acetyl Novolac			

**Table 3:** The composition of the organic phases studied in Section 3.2. All phases also contain 15 mL of cyclohexanone.

Mixture Code	Type of Resin	Resin				MDI (wt.%) (mmol NCO)	IPDI (wt.%) (mmol NCO)	HDI (wt.%) (mmol NCO)
		(wt.%)	Total OH (mmol)	Phenolic OH (mmol)	Aliphatic OH (mmol)			
I-NOV	Novolac resin	19.8	65	43	22	4.0 wt.%	19.8 wt.%	-
I-BNZ	Benzyl Novolac resin		11.8	-	11.8			
I-AC	Acetyl Novolac resin		-	-	-	(8 mmol)	(44 mmol)	
H-NOV	Novolac resin	21.2	65	43	22	4.2 wt.%	-	14.2 wt.%
H-BNZ	Benzyl Novolac resin		11.8	-	11.8			
H-AC	Acetyl Novolac resin		-	-	-	(8 mmol)		(40 mmol)

## Data Availability

Data will be made available on request.

## Supplementary Materials

The following supporting information can be downloaded at: https://www.mdpi.com/article/10.3390/polym16020270/s1, Table S1. Results of titrations for the three types of HDI-loaded MCs using Equations (S1) and (S3). The titration procedure was achieved around 45 days after MCs formation; Figure S1: Visual observation of H-NOV, I-NOV, H-BNZ, I-BNZ, H-AC and I-AC mixtures just after mixing (t0) and 1 or 14 days later; Figure S2: Polyurea microcapsules using Novolac resin loaded with (A) IPDI and (B) HDI; Figure S3: Evolution of ATR-FTIR spectra of (A) I-BNZ (B) H-BNZ mixtures with time; Figure S4: Evolution of ATR-FTIR spectra of (A) I-AC (B) H-AC mixtures with time.
