# Supplementary material for: Controlling the Synthesis of Polyurea Microcapsules and the Encapsulation of Active Diisocyanate Compounds
Source: Polymers (Basel). 2024 Jan 18;16(2):270. doi: 10.3390/polym16020270 (PMC10820442; doi:10.3390/polym16020270)
Supplement: Supplementary file 1 [file polymers-16-00270-s001.zip › polymers-2812285-supplementary.pdf]

Supplementary Notes

S1. Determination of relative percentage of the NCO compound

For the determination of NCO content in the study of organic phases, equation 1 is used, while for the determination of the encapsulated diisocyanate compound (DI) equations S2 and S3 are used in combination with equation S1.

$$NCO, \% = \frac{(V_{blank} - V) x\, N_{HCl}\, x\, 0.042}{M}\, x\, 100 \quad (\boldsymbol{S1})$$

$$m_{DI_{release}} = \frac{2\, x\, \frac{NCO, \%}{2\, x\, 42}\, x\, Mr_{DI}}{100 - 2\, x\, \frac{NCO, \%}{2\, x\, 42}\, x\, Mr_{DI}}\, x\, m_{solvent} \quad (\boldsymbol{S2})$$

$$DI_{wt.\%} = \frac{m_{DI_{release}}}{m_{DI_{encapsulated}}}\, x\, 100 \quad (\boldsymbol{S3})$$

where NCO, % is the NCO content in an organic solvent, $m_{\boldsymbol{DI}\text{release}}$ is the mass of released diisocyanate (DI) of MCs and $DI_{\mathbf{wt\%}}$ is the percentage of DI released from MCs. Furthermore, V_{blank} (mL) and V (mL) are the volumes of the standard HCl aqueous solution consumed during the blank experiment and sample titration, respectively. N_{HCl} is the normality of standard HCl aqueous solution, 0.042 is the milliequivalent weight of the NCO group, Mr_{DI} is the molar mass of DI and M (g), $m_{solvent}$ (g) and $m_{DIencapsulated}$ (g) are the masses of the sample, organic solvent and encapsulated DI (considering the feed composition of the mixture for the synthesis of MCs), respectively.

The results obtained from the above equations for the core material of HDI-loaded microcapsules are presented in **Table S1**.

Table S1. Results of titrations for the three types of HDI-loaded MCs using Eqs. (1) and (3). The titration procedure was achieved around 45 days after MCs formation.

HDI-loaded MCs with	NCO, %	HDI, wt.%
Acetyl Novolac resin	0.21	90.6
Benzyl Novolac resin	0.05	20.3
Novolac resin	0.04	18.9

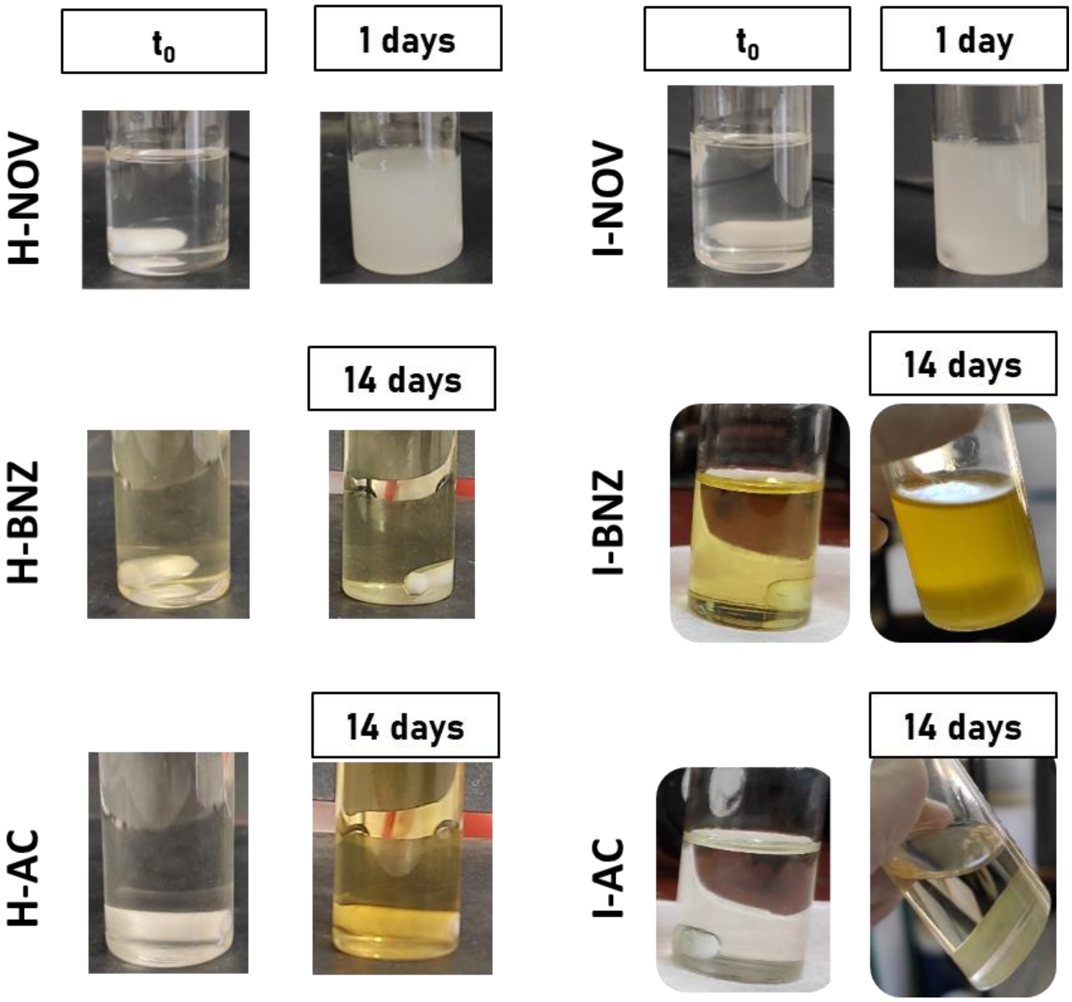

Figure S1: Visual observation of H-NOV, I-NOV, H-BNZ, I-BNZ, H-AC and I-AC mixtures just after mixing (t_0) and 1 or 14 days later.

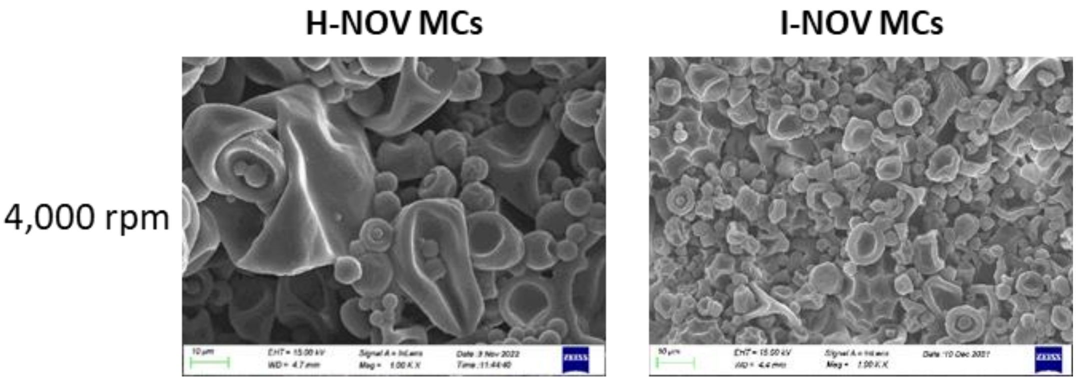

Figure S2: Polyurea microcapsules using Novolac resin loaded with (A) IPDI and (B) HDI.

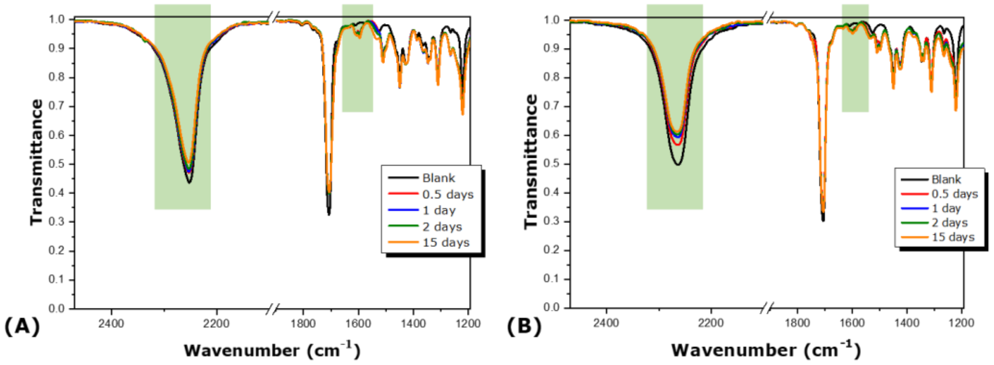

Figure S3: Evolution of ATR-FTIR spectra of (A) I-BNZ (B) H-BNZ mixtures with time.

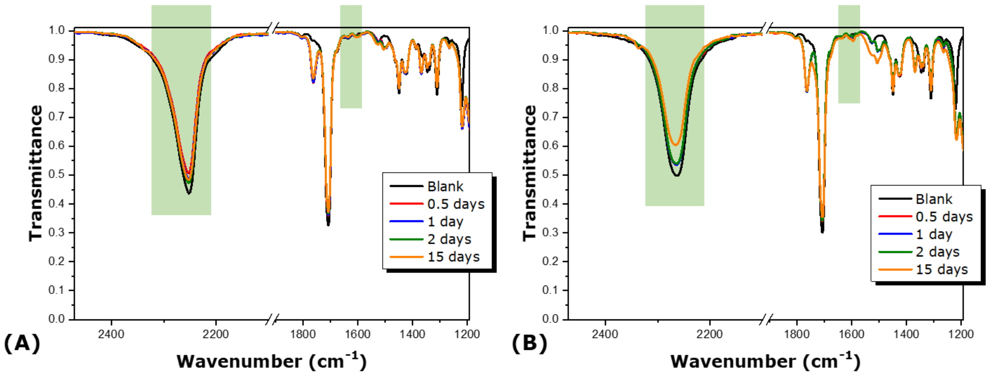

Figure S4: Evolution of ATR-FTIR spectra of (A) I-AC (B) H-AC mixtures with time.
